# Frequency-domain attention enhanced YOLOv11-EfficientFormerV2 for Tiny lesion detection in complex field plant images

**DOI:** 10.3389/fpls.2026.1876223

**Published:** 2026-07-03

**Authors:** Shanjiang Zhang, Renjing Liu

**Affiliations:** School of Management, Xi’an Jiaotong University, Xi’an, China

**Keywords:** EfficientFormerV2, frequency-domain attention, plant disease lesion detection, PlantDoc dataset, Tiny lesion, YOLOv11

## Abstract

**Introduction:**

To address the challenges of low detection precision, severe background interference, and high model complexity in tiny crop disease lesion detection (defined as lesions occupying 8×8 to 32×32 pixels at 640×640 input resolution) under complex field environments, this study proposes a lightweight detection model named FDA-YOLO by integrating frequency-domain attention and improved YOLOv11.

**Methods:**

The model employs EfficientFormerV2 as the backbone to extract multi-scale features with low computational cost, and introduces a frequency domain attention module to enhance high-frequency tiny disease lesion details and suppress background noise.

**Results:**

Comprehensive experiments on the PlantDoc dataset demonstrate that the proposed model achieves 96.3% mAP@0.5, 96.8% precision, and 36.4 FPS with only 28.5M parameters, outperforming the selected baseline detectors under the adopted experimental setting.

**Discussion:**

The model realizes an optimal balance between accuracy, efficiency, and lightweight performance, providing a reliable and practical solution for real-time tiny lesion detection inprecision agriculture and edge device deployment.

## 1 Introduction

Precision agriculture (Sharma and Angidi, 2022) has become an indispensable direction for modern agricultural development, and real-time, high-precision detection of crop disease lesions is a critical prerequisite for intelligent field management, disease early warning, and yield estimation (Raj and Prahadeeswaran, 2025). With the rapid advancement of computer vision (Khatri and Shinde, 2022) and deep learning (Cao et al., 2025) technologies, data-driven object detection algorithms have gradually replaced traditional manual inspection methods, providing efficient and reliable technical support for crop health monitoring (Liu et al., 2025). However, in real field environments, automated plant disease lesion detection still faces numerous severe challenges that restrict detection performance and practical deployment.

Field plant images are commonly disturbed by complex and changeable backgrounds, including soil, weeds, shadows, and light reflections (Moupojou et al., 2023), which cause serious interference with disease lesion feature extraction. Meanwhile, most early crop disease lesions are tiny and sparse (typically occupying 8×8 to 32×32 pixels at 640×640 input resolution), usually occupying only a few pixels in the image with extremely low contrast against the surrounding environment (Yang et al., 2020). Such tiny lesions are prone to being submerged in background noise during feature extraction, leading to a high missed detection rate. In addition, practical agricultural detection systems require deployment on edge devices such as unmanned aerial vehicles and field robots, which have limited computing resources and impose strict constraints on model parameters, computational complexity, and inference speed. Therefore, developing a lightweight detection model with high accuracy for tiny disease lesions has become an urgent demand in precision agriculture.

Traditional convolutional neural network (CNN)-based detection models, represented by Faster RCNN (Ren et al., 2017), rely on convolutional operations for local feature perception and have achieved remarkable progress in various detection tasks (Arulprakash et al., 2025). Nevertheless, the inherent locality of convolution limits the long-range context modeling ability of these models, making it difficult to establish effective global relationships between tiny disease lesions and complex backgrounds. Moreover, repeated downsampling operations in deep CNNs lead to the loss of high-frequency details of tiny lesions, further reducing detection sensitivity. Although some improved CNN models with enhanced attention mechanisms (Hu et al., 2018; Woo et al., 2018; Wang et al., 2020) have alleviated this problem to a certain extent, the contradiction between local perception bias and tiny lesion feature preservation still cannot be fundamentally solved (El Sakka et al., 2025).

The emergence of vision transformer models (Han et al., 2023) breaks the limitations of traditional CNNs by capturing long-range global context information through self-attention mechanisms (Vaswani et al., 2017), providing a new solution for global feature modeling. Representative transformer-based detectors such as ViT (Dosovitskiy et al., 2021) and Swin Transformer (Liu et al., 2021) have demonstrated powerful feature representation capabilities on large-scale datasets. However, these models rely on massive computing resources and have huge parameters and computational overheads, which are not conducive to practical deployment on agricultural edge devices with limited performance. Furthermore, pure transformer models still lack sufficient sensitivity to high-frequency tiny lesion details in the spatial domain, resulting in limited performance improvement for tiny plant disease lesion detection.

To address the aforementioned challenges, this study proposes a lightweight and high-performance detection model named FDA-YOLO, which integrates frequency-domain attention and lightweight transformer architecture into the YOLOv11 framework (Khanam and Hussain, 2024c). The core motivation of this study is to enhance the representation of tiny lesion features from the perspective of frequencydomain information while achieving efficient inference through a lightweight transformer backbone. Different from traditional spatial-domain enhancement methods, the proposed frequency-domain attention mechanism operates in the frequency domain to generate channel-wise adaptive weights based on spectral magnitude statistics. By modulating the amplitude spectrum of each channel, the FDA module strengthens channels that contain lesion-relevant frequency content while attenuating channels dominated by background noise, thereby improving the representation of tiny lesion features. Meanwhile, the lightweight EfficientFormerV2 (Li et al., 2022, Li et al., 2023) backbone ensures global context modeling ability with low computational cost, balancing detection accuracy and model efficiency.

The main contributions of this study are summarized as follows. First, a novel frequency-domain attention module is proposed to enhance high-frequency tiny lesion features, which effectively suppresses complex background interference and significantly reduces the missed detection rate of tiny lesions. Second, the lightweight EfficientFormerV2 is introduced as the backbone to replace the original CNN structure, reducing model parameters and computational complexity while maintaining strong feature extraction ability. Third, the proposed FDA-YOLO achieves an optimal balance among detection accuracy, inference speed, and model lightweightness on the public PlantDoc dataset (Singh et al., 2020), exhibiting superior comprehensive performance compared with mainstream CNN-based and transformer-based detectors under the same experimental setting. Fourth, a comprehensive ablation study and comparative analysis with existing attention mechanisms including SE (Hu et al., 2018), CBAM (Woo et al., 2018), ECA (Wang et al., 2020), and FcaNet (Qin et al., 2021) are conducted to validate the unique advantages of the proposed FDA module in frequency-domain feature enhancement for tiny lesion detection.

Extensive experimental results verify that the proposed model not only improves the detection accuracy of tiny lesions but also meets the efficiency requirements of practical agricultural applications. The rest of this paper is organized as follows: Section 2 reviews the related work on plant disease detection and attention mechanisms. Section 3 introduces the detailed methodology of the proposed FDA-YOLO model. Section 4 presents the experimental setup, implementation details, and comprehensive experimental results and analysis. Section 5 discusses the rationality, effectiveness, and limitations of the proposed method. Section 6 concludes the whole study and prospects future research directions.

## 2 Related work

### 2.1 Deep learning for plant disease detection

The application of deep learning in plant disease detection has evolved significantly over the past decade, transitioning from laboratory-controlled settings to challenging real-world field conditions (Mohanty et al., 2016; Ferentinos, 2018). Early seminal work by Mohanty et al. (2016) demonstrated that deep CNNs could classify 14 crop species and 26 diseases with over 99% accuracy on the PlantVillage dataset, establishing deep learning as a viable approach for automated plant disease diagnosis. Subsequent studies have extended this direction by exploring various architectures including AlexNet, VGG, GoogLeNet, and ResNet for plant disease classification (Ferentinos, 2018; Too et al., 2019).

However, a critical limitation of these classification-based approaches is that they assume isolated leaf images captured under controlled conditions and only output category labels without localization information (Barbedo, 2018; Luo et al., 2024). In practical agricultural scenarios, field images contain complex backgrounds with multiple leaves, overlapping objects, varying illumination, and diverse symptom manifestations (Moupojou et al., 2023). Therefore, object detection frameworks that can simultaneously classify and localize disease lesions have gained increasing attention. Fuentes et al. (2017); Karthik et al. (2024) pioneered the application of object detection algorithms for tomato disease and pest recognition in complex field images. Singh et al. (2020) introduced the PlantDoc dataset specifically for object-level plant disease detection, featuring 2,598 field-captured images with bounding box annotations covering 13 crop species and 27 disease categories. Recent studies have applied various YOLO variants (Khanam and Hussain, 2024a, Khanam and Hussain, 2024b, Khanam and Hussain, 2024c) and transformer-based detectors (Dosovitskiy et al., 2021; Liu et al., 2021) on the PlantDoc dataset, yet the detection of tiny, low-contrast disease lesions in complex field environments remains a challenging problem.

### 2.2 Attention mechanisms in visual detection

Attention mechanisms have become essential components in modern visual detection systems by enabling models to focus on informative regions while suppressing irrelevant background responses (Hu et al., 2018). The seminal Squeeze-and-Excitation (SE) attention (Hu et al., 2018) introduced channel-wise recalibration by explicitly modeling interdependencies between channels, significantly improving the representational capacity of CNNs. Woo et al. (2018) proposed the Convolutional Block Attention Module (CBAM) that sequentially applies channel and spatial attention to refine feature maps at both dimensions. Wang et al. (2020) developed Efficient Channel Attention (ECA) that avoids dimensionality reduction and achieves competitive performance with lower computational cost through local cross-channel interaction.

Beyond these spatial-domain attention methods, frequency-domain attention has emerged as a promising direction for enhancing fine-grained feature representation. Qin et al. (2021) proposed FcaNet that extends channel attention to the frequency domain by incorporating discrete cosine transform (DCT) coefficients, demonstrating that frequency-domain information can effectively complement spatial-domain features. Wavelet-based attention methods (Liu and Wang, 2026) have also been explored for decomposing features into different frequency sub-bands and applying attention selectively. However, existing frequency-domain attention methods primarily focus on channel-level global pooling and do not explicitly exploit the spatialfrequency correspondence for tiny target enhancement in object detection tasks. The proposed FDA module addresses this gap by directly operating on the 2D frequency spectrum to enhance high-frequency components corresponding to tiny disease lesions while suppressing low-frequency background noise.

### 2.3 Lightweight vision transformers for edge deployment

The deployment of high-performance detection models on resource-constrained agricultural edge devices has motivated extensive research on lightweight architectures. While standard vision transformers such as ViT (Dosovitskiy et al., 2021) and Swin Transformer (Liu et al., 2021) achieve impressive accuracy, their quadratic computational complexity with respect to sequence length makes them unsuitable for real-time edge applications (Li et al., 2022). To address this limitation, various efficient transformer architectures have been proposed, including MobileViT (Mehta and Rastegari, 2021), PVT (Wang et al., 2021), and EfficientFormer (Li et al., 2022).

EfficientFormerV2 (Li et al., 2023) represents a significant advancement in this direction by rethinking the architecture design for MobileNet-level size and speed. Through optimized token mixing, improved attention mechanisms, and hardware-aware design choices, EfficientFormerV2 achieves strong performance on visual tasks while maintaining computational efficiency suitable for edge deployment. This study adopts EfficientFormerV2 as the backbone network to replace the original CNN structure in YOLOv11, aiming to leverage the global context modeling capability of transformers without compromising the efficiency requirements of agricultural edge devices.

## 3 Methodology

### 3.1 Overall framework and design motivation

**Figure 1:**
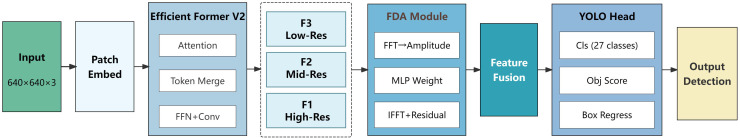
Detailed architecture of the proposed Frequency-Domain Attention YOLO (FDA-YOLO). The framework consists of four key components: the input layer, a lightweight EfficientFormerV2 backbone for multi-scale feature extraction, a Frequency-Domain Attention (FDA) module for enhancing tiny lesion features, and an improved YOLOv11 detection head for classification (Cls), objectness score (Obj), and bounding box regression (Box). The model realizes end-to-end tiny crop disease lesion detection. EfficientFormerV2-S0 variant is used with output stages producing features at resolutions corresponding to P3 (80×80), P4 (40×40), and P5 (20×20) levels when the input resolution is 640×640.

In real agricultural field environments, images captured by imaging equipment often contain complex and heterogeneous backgrounds, while early crop disease spots and phenotypic lesions are characterized by tiny sizes, low contrasts, and unobvious texture features. Such characteristics lead to the high-frequency components of tiny lesions being easily ignored or lost in traditional spatial-domain convolution operations, resulting in a high missed detection rate. Meanwhile, the deployment requirements of edge computing devices impose strict constraints on model parameters and computational complexity. To address these problems, this study proposes an end-to-end lightweight detection network named FDA-YOLO, which is designed for tiny crop disease lesion detection tasks under complex backgrounds. The overall architecture of the proposed model is illustrated in **Figure 1**. The framework consists of three core components: a lightweight EfficientFormerV2 backbone for multi-scale feature extraction, a frequency-domain attention (FDA) module for enhancing high-frequency tiny lesion features, and an improved YOLOv11 detection head for lesion classification and localization. The core design philosophy is to enhance the expression of tiny lesion features in the frequency domain while maintaining efficient model inference, so as to achieve a reasonable balance between detection accuracy, computational efficiency, and lightweight performance.

### 3.2 Lightweight backbone based on EfficientFormerV2

Traditional CNN-based backbones have limited ability to capture global context information, while standard Transformer architectures introduce huge computational overhead, which is not suitable for agricultural edge deployment. In this study, the lightweight EfficientFormerV2-S0 (Li et al., 2023) is used as the feature extraction backbone, which maintains global modeling capability while significantly reducing computational complexity and the number of parameters. This backbone is particularly suitable for extracting multi-scale features of crop disease lesions with large-scale variations.

Specifically, EfficientFormerV2-S0 consists of four stages with hierarchical feature dimensions. When the input image resolution is 640×640×3, the backbone produces multi-scale outputs at four stages with spatial resolutions of 160×160 (stage 1), 80×80 (stage 2), 40×40 (stage 3), and 20×20 (stage 4), with corresponding channel dimensions of 32, 48, 128, and 192, respectively. The outputs from stages 2, 3, and 4 are mapped to the P3, P4, and P5 levels of the YOLOv11 neck, respectively, maintaining the PAN/FPN-based feature fusion structure. The detailed architecture specifications are summarized in **Table 1**.

**Table 1:** EfficientFormerV2-S0 backbone architecture specifications for input size 640×640.

Stage	Resolution	Channels	YOLO level	Key operations
Stem	320×320	32	–	Overlap Patch Embed
1	160×160	32	–	PoolFormer Block ×2
2	80×80	48	P3	Attention Block ×2
3	40×40	128	P4	Attention Block ×4
4	20×20	192	P5	Attention Block ×3

Given an input field plant image $f\in {\mathbb{R}}^{H\times W\times 3}$ used for disease lesion detection, the patch embedding layer converts the image into a sequence of visual tokens suitable for Transformer computing, which can be expressed as:

(1) $$T_{0}=\text{PatchEmbed }(f)\in {\mathbb{R}}^{N\times C}$$

where *H* and *W* represent the height and width of the input disease lesion image, 3 denotes the number of RGB channels, *N* represents the number of visual tokens, and *C* represents the feature dimension of each token. As described in **Equation 1**, this operation converts pixel-level raw image information into structured features, laying the foundation for subsequent global context modeling of disease lesion targets.

The core of EfficientFormerV2 is the meta-dimension self-attention mechanism, which captures longrange dependencies between tiny disease lesions and background regions. The self-attention calculation process is defined as:

(2) $$\text{Attention }(Q,K,V)=\text{Softmax }(\frac{QK^{T}}{\sqrt{C}})V$$

where *Q*, *K*, and *V* represent query, key, and value matrices generated by linear projection of visual tokens. As shown in **Equation 2**, this mechanism can establish global semantic associations for tiny disease spots, thereby improving the ability to distinguish lesions from complex backgrounds.

To reduce the number of tokens and computational redundancy, the token merging operation is used to downsample the feature sequence:

(3) $$T_{l+1}=\text{TokenMerge }(T_{l})\in {\mathbb{R}}^{\frac{N}{4}\times 2C}$$

where *T_l_* represents the input token feature, and *T_l_*_+1_ represents the output after merging. According to **Equation 3**, this operation reduces the number of tokens by 75% while expanding the channel dimension, which effectively improves the efficiency of multi-scale disease lesion feature extraction.

The feed-forward network (FFN) enhances the local sensing ability of disease lesion features by introducing depth-wise convolution:

(4) FFN (x)=Conv (GELU (Linear (x)))

where *x* represents the input token feature, and GELU is the activation function. As illustrated in **Equation 4**, this hybrid structure combines the global modeling ability of Transformer and the local detail extraction ability of CNN, which is conducive to capturing fine-grained features of tiny disease lesions.

After multi-stage feature extraction, the backbone outputs a multi-scale feature pyramid containing rich disease lesion information:

(5) $$F=\{F_{1},F_{2},F_{3}\}$$

where *F*_1_, *F*_2_, and *F*_3_ represent high-resolution (P3, 80×80), medium-resolution (P4, 40×40), and lowresolution (P5, 20×20) disease lesion feature maps respectively when the input is 640×640. As shown in **Equation 5**, the multi-scale feature pyramid can adapt to lesions of different sizes in the PlantDoc dataset, especially improving the detection sensitivity of tiny targets.

### 3.3 Frequency-domain attention module for tiny lesion enhancement

**Figure 2:**
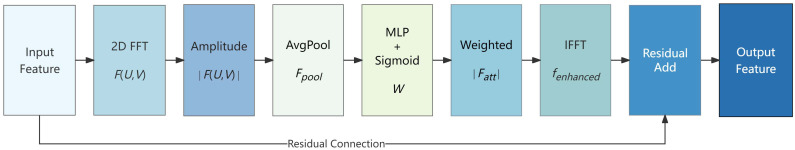
Detailed internal structure of the proposed Frequency-Domain Attention (FDA) module. The pipeline includes: 2-Dimensional Fast Fourier Transform (2D FFT), amplitude spectrum extraction, average pooling (AvgPool), multi-layer perceptron (MLP) attention generation, weighted frequency enhancement, inverse fast Fourier transform (IFFT), and residual connection for feature fusion and stability training. The FDA module operates on each channel independently and preserves the original phase information during frequency-domain processing.

Tiny crop disease lesions correspond to high-frequency components in the frequency domain, which are easily lost in spatial-domain convolution and downsampling. To solve this problem, a frequency-domain attention (FDA) module is designed to enhance high-frequency lesion details and suppress low-frequency background noise. The detailed structure of the FDA module is visually presented in **Figure 2**.

#### 3.3.1 Module insertion position and tensor flow

The FDA module is applied to the P4-level feature map (*F*_2_, 40×40×128) in the backbone output pyramid. This choice is motivated by the observation that tiny disease lesions typically occupy 8×8 to 32×32 pixel regions in the 640×640 input images, and the P4-level feature map provides an optimal balance between spatial resolution and semantic richness for detecting such small targets. The P3-level features have higher resolution but insufficient semantic information, while the P5-level features have rich semantics but too low resolution for tiny lesions.

Given an input feature map $X\in {\mathbb{R}}^{B\times C\times H\times W}$, where *B* is the batch size, *C* is the number of channels, and *H*, *W* are spatial dimensions, the FDA module processes each channel independently. Specifically, for each channel *c* ∈ {1,2*,…,C*}, the 2D FFT is applied separately:

(6) $$F_{c}(u,v)=\sum_{x=0}^{H-1}\sum_{y=0}^{W-1}X_{c}(x,y)\cdot e^{-2\pi i(ux/H+vy/W)}$$

where *X_c_*(*x,y*) represents the spatial feature map of channel *c*, and *F_c_*(*u,v*) represents the frequency spectrum of channel *c*. Note that the upper limit of the second summation is (*W* − 1), correcting a dimensional error in the previous formulation. According to **Equation 6**, this transformation can separate high-frequency tiny lesion components from low-frequency background components on a per-channel basis, enabling targeted feature enhancement.

#### 3.3.2 Amplitude-phase decomposition and attention generation

The frequency spectrum is decomposed into amplitude and phase components:

(7) $$F_{c}(u,v)=|F_{c}(u,v)|\cdot e^{i∠F_{c}(u,v)}$$

where |*F_c_*(*u,v*)| represents the amplitude spectrum reflecting the intensity of disease lesion features, and ∠*F_c_*(*u,v*) represents the phase spectrum containing the structural information. As shown in **Equation 7**, the amplitude contains most of the energy distribution information of tiny lesions, so the FDA module performs adaptive weighting on the amplitude while preserving the original phase information.

Global average pooling is used to extract global frequency distribution information:

(8) $$F_{\text{pool},c}=\text{AvgPool }(|F_{c}(u,v)|)=\frac{1}{H\times W}\sum_{u=0}^{H-1}\sum_{v=0}^{W-1}|F_{c}(u,v)|$$

where |*F_c_*(*u,v*)| is the amplitude spectrum of channel *c*. According to **Equation 8**, this operation compresses the global frequency information into a channel-wise statistic, providing a basis for generating adaptive attention weights.

A lightweight MLP with a hidden dimension of *C/*16 generates attention weights to enhance highfrequency lesion components. The MLP structure consists of two fully-connected layers with a reduction ratio *r* = 16:

(9) $$W_{c}=\sigma (\text{MLP }(F_{\text{pool},c}))=\sigma (W_{2}\cdot \text{ReLU }(W_{1}\cdot F_{\text{pool},c}))$$

where *σ* is the sigmoid activation function that limits the weight range between 0 and 1, $W_{1}\in {\mathbb{R}}^{C/r\times C}$ and $W_{2}\in {\mathbb{R}}^{C\times C/r}$ are the MLP weight matrices. The hidden dimension is set to *C/*16 (e.g., 8 for *C* = 128) to minimize parameter overhead. As shown in **Equation 9**, the generated channel-wise weight scalar *W_c_* modulates the frequency spectrum of each channel to enhance components related to lesions and suppress background noise.

#### 3.3.3 Frequency-domain enhancement and reconstruction

The attention weight is multiplied with the original amplitude spectrum to achieve frequency-domain enhancement:

(10) $$|F_{\text{att},c}(u,v)|=|F_{c}(u,v)|\cdot W_{c}$$

where · represents scalar multiplication applied uniformly across all frequency positions (*u,v*) for channel *c*. According to **Equation 10**, channels with higher attention weights will have their entire frequency spectrum amplified, while channels with lower weights will be attenuated. This channel-wise frequency modulation strengthens the expression of tiny lesion features and reduces the interference of complex field backgrounds.

The enhanced frequency spectrum is then reconstructed by combining the modulated amplitude with the original preserved phase:

(11) $${\hat{F}}_{c}(u,v)=|F_{\text{att},c}(u,v)|\cdot e^{i∠F_{c}(u,v)}$$

where $e^{i∠F_{c}(u,v)}$ is the original phase information. As shown in **Equation 11**, preserving the original phase ensures that the spatial structural information of the input features is maintained during frequency-domain processing, preventing distortion of the reconstructed features.

The inverse Fourier transform converts the enhanced frequency-domain features back to the spatial domain:

(12) $${\hat{X}}_{c}(x,y)=\frac{1}{H\times W}\sum_{u=0}^{H-1}\sum_{v=0}^{W-1}{\hat{F}}_{c}(u,v)\cdot e^{2\pi i(ux/H+vy/W)}$$

where ${\hat{X}}_{c}(x,y)$ is the real part of the inverse-transformed output (the imaginary part is negligible due to Hermitian symmetry of the modified spectrum). As shown in **Equation 12**, this step reconstructs the spatial features with enhanced tiny lesion details.

A residual connection is used to fuse the original features and enhanced features:

(13) $$F_{\text{enhanced}}=\text{BN }(\sum_{c=1}^{C}{\hat{X}}_{c}+X)$$

where *X* is the input feature of the FDA module. According to **Equation 13**, the residual connection ensures the stability of model training and retains the original context information while enhancing lesion features.

#### 3.3.4 Comparison with existing attention mechanisms

The proposed FDA module differs from existing attention mechanisms in several key aspects. Unlike SE attention (Hu et al., 2018) and ECA (Wang et al., 2020) that operate entirely in the spatial domain using global average pooling, FDA transforms features into the frequency domain to exploit spectral statistics for channel-wise recalibration. Compared to FcaNet (Qin et al., 2021) that uses DCT-based channel attention for image classification, FDA employs 2D FFT for object detection and preserves phase information during frequency-domain processing. Unlike CBAM (Woo et al., 2018) that applies separate channel and spatial attention modules, FDA integrates frequency-domain analysis in a unified lightweight module with fewer parameters. It should be noted that FDA performs channel-wise amplitude modulation rather than explicit high/low/mid-frequency band selection; the learned channel weights implicitly favor channels containing lesion-relevant frequency content. The parameter count of FDA is only (*C* × *C/r* + *C/r* × *C*) = 2*C*^2^*/r* (e.g., 2,048 parameters for *C* = 128*,r* = 16), which is significantly smaller than CBAM and comparable to SE attention.

### 3.4 Improved YOLOv11 detection head and loss function

The enhanced multi-scale features are fed into the improved YOLOv11 detection head to complete disease lesion classification and bounding box regression for the PlantDoc dataset. Following the YOLOv11 anchor-free design, the detection head directly predicts bounding box coordinates without relying on predefined anchor boxes. The regression branch employs Distribution Focal Loss (DFL) for fine-grained bounding box location estimation, which treats bounding box coordinates as distributions rather than deterministic values.

The classification loss uses binary cross-entropy to distinguish 27 types of crop disease lesions and background regions:

(14) $$L_{\text{cls}}=-\frac{1}{N}\sum_{i=1}^{N}[y_{i}\log\;(p_{i})+(1-y_{i})\log\;(1-p_{i})]$$

where *y_i_*is the ground-truth label of the disease lesion category, *p_i_*is the predicted category probability, and *N* is the number of sampling points. As shown in **Equation 14**, this loss ensures accurate classification of various crop disease lesions.

The bounding box regression loss uses CIoU combined with DFL to improve the localization accuracy of tiny lesions:

(15) $$L_{\text{box}}=\lambda_{\text{cio}}L_{\text{CIoU}}+\lambda_{\text{dfl}}L_{\text{DFL}}$$

where $L_{\text{CIoU}}=1-\text{CIoU }(B,\hat{B})$ with *B* being the ground-truth box and $\hat{B}$ being the predicted box, and $L_{\text{DFL}}$ is the Distribution Focal Loss for bounding box coordinate regression. According to **Equation 15**, this combined loss considers the overlap area, center distance, aspect ratio, and coordinate distribution, which effectively improves the positioning accuracy of tiny disease spots.

The objectness loss distinguishes between disease lesion targets and non-target backgrounds:

(16) $$L_{\text{obj}}=-\frac{1}{N}\sum_{i=1}^{N}[{\hat{y}}_{i}\log\;({\hat{p}}_{i})+(1-{\hat{y}}_{i})\log\;(1-{\hat{p}}_{i})]$$

where ${\hat{y}}_{i}$is the objectness label, and ${\hat{p}}_{i}$is the predicted objectness score. As shown in **Equation 16**, this loss reduces false detection caused by complex backgrounds.

The total loss function jointly optimizes classification, regression, and objectness:

(17) $$L_{\text{total}}=\lambda_{1}L_{\text{cls}}+\lambda_{2}L_{\text{box}}+\lambda_{3}L_{\text{obj}}$$

where *λ*_1_ = 0.5, *λ*_2_ = 7.5, and *λ*_3_ = 1.0 are the balance coefficients empirically determined through validation experiments. The higher weight for *λ*_2_ reflects the importance of accurate localization for tiny lesions. According to **Equation 17**, the joint loss ensures stable model optimization and improves the overall detection performance of crop disease lesions.

During inference, non-maximum suppression removes redundant boxes:

(18) $$B_{\text{final}}=\text{NMS }(B_{\text{pred}},\tau )$$

where $B_{\text{pred}}$ is the initial prediction set, and *τ* = 0.7 is the IoU threshold. As shown in **Equation 18**, this step retains the most reliable disease lesion detection results.

### 3.5 Multi-scale feature fusion and lesion prediction

To further improve the detection performance of multi-scale disease lesions, a feature fusion strategy is adopted. The neck structure maintains the PAN/FPN architecture with C2f and SPPF modules from YOLOv11:

(19) $$F_{\text{fusion}}=\text{Concat }(F_{\text{up}1},F_{\text{conv}1},F_{\text{up}2},F_{\text{conv}2})$$

where *F*_up1_ and *F*_up2_ are upsampled features, and *F*_conv1_ and *F*_conv2_ are convolved features. According to **Equation 19**, this operation integrates high-resolution details and high-level semantics.

Finally, multi-scale prediction is performed to output the final disease lesion detection results at three scales:

(20) $$\hat{Y}=\{{\hat{Y}}_{\text{P}3},{\hat{Y}}_{\text{P}4},{\hat{Y}}_{\text{P}5}\}=\text{Head }(F_{\text{fusion}})$$

where ${\hat{Y}}_{\text{P}3}$, ${\hat{Y}}_{\text{P}4}$, and ${\hat{Y}}_{\text{P}5}$ are prediction results at P3 (80×80), P4 (40×40), and P5 (20×20) scales respectively. As shown in **Equation 20**, the model can accurately detect tiny and large-scale crop disease lesions simultaneously.

## 4 Experiments

### 4.1 Experimental setup and implementation details

All experiments are conducted on the public PlantDoc dataset (Singh et al., 2020) for plant disease and leaf-condition detection, which contains 2,598 RGB images covering 13 crop species and 27 plant disease and leaf-condition categories. PlantDoc provides bounding box annotations for object-level disease lesion detection, making it suitable for evaluating the proposed method. The dataset is randomly divided into training and testing sets with an 8:2 ratio (2,078 training images and 520 testing images) using a fixed random seed of 42 for reproducibility. Prior to splitting, a duplicate image check is performed using perceptual hashing (pHash) to identify and remove potential near-duplicate images across the dataset, thereby avoiding data leakage between training and testing sets. All input images are uniformly resized to 640×640 pixels to ensure consistency during training and inference. The hardware platform consists of an Intel Core i9-12900K CPU and an NVIDIA GeForce RTX 3090 GPU with 24GB memory, and the operating system is Ubuntu 22.04. The whole framework is built on the PyTorch 2.0 (Paszke et al., 2019) deep learning framework with CUDA 11.8 and Python 3.9.

To address class imbalance in the PlantDoc dataset, which contains varying numbers of samples across the 27 plant disease and leaf-condition categories, images with fewer samples receive higher sampling weights during training. Additionally, Mosaic augmentation is applied with a probability of 0.5, and the copy-paste augmentation for tiny lesions is used with a probability of 0.3 to increase the representation of small targets. All models are trained for 100 epochs under the same environment to ensure fair comparison.

To ensure stable convergence and reliable detection performance, a complete set of training configurations is adopted. The Stochastic Gradient Descent (SGD) optimizer is utilized for network optimization, with a fixed batch size and appropriate learning rate scheduling strategy. Data augmentation strategies including random horizontal flipping (probability 0.5), random rotation within 10 degrees, multi-scale scaling (range 0.5 to 1.5), and Mosaic augmentation are applied to improve the generalization ability and robustness of the model. The detailed experimental parameters and configurations are listed in **Table 2**.

**Table 2:** Detailed experimental parameters and configurations.

Parameter	Value
Input Image Size	640×640
Training Epochs	100
Batch Size	32
Optimizer	SGD
Initial Learning Rate	0.01
Momentum	0.937
Weight Decay	5e-4
Learning Rate Scheduler	Cosine Annealing
$\lambda_{1}$ (Classification)	0.5
$\lambda_{2}$ (Regression)	7.5
$\lambda_{3}$ (Objectness)	1.0
NMS IoU Threshold	0.7
Confidence Threshold	0.25
Data Augmentation	Flip, Rotation, Scaling, Mosaic
Random Seed	42
Train/Test Split	2,078/520
GPU Device	NVIDIA RTX 3090 (24GB)
Deep Learning Framework	PyTorch 2.0

To comprehensively evaluate model performance, six mainstream evaluation metrics are adopted, including Precision (P), Recall (R), mean Average Precision at IoU=0.5 (mAP@0.5), mAP@0.5:0.95, Frames Per Second (FPS), Parameters (Params), and GFLOPs. To ensure statistical reliability, all reported results are the mean and standard deviation across three independent training runs with different random seeds (42, 123, and 456). These metrics cover detection accuracy, inference efficiency, and model complexity, enabling a full-dimensional evaluation of the proposed method.

### 4.2 Comparison with state-of-the-art models

To verify the superiority and advancement of the proposed FDA-YOLO model, comprehensive comparison experiments are conducted with various representative deep learning models. These models include classic CNN-based detectors and backbone networks such as Faster R-CNN (Ren et al., 2017), ResNet50-FPN (He et al., 2015), and EfficientNetB2 (Tan and Le, 2019), representative Transformer-based models such as ViT-B/16 (Han et al., 2023) and Swin-T (Liu et al., 2021), as well as the latest YOLO series detectors including YOLOv5 (Khanam and Hussain, 2024a), YOLOv8 (Khanam and Hussain, 2024b), and YOLOv11 (Khanam and Hussain, 2024c). All comparison models are trained and tested under the same experimental environment and dataset split to ensure fair and objective comparison results.

The quantitative comparison results of all models are shown in **Table 3**. It can be observed that the proposed FDA-YOLO model achieves the best comprehensive performance. Specifically, our model obtains 96.8 ± 0.2% Precision, 95.7 ± 0.3% Recall, 96.3 ± 0.2% mAP@0.5, and 72.1 ± 0.4% mAP@0.5:0.95, which are significantly higher than all CNN-based and Transformer-based comparison models. In terms of lightweight performance and inference efficiency, FDA-YOLO only has 28.5M parameters and 41.2 GFLOPs, and the inference speed reaches 36.4 FPS. The results demonstrate that the proposed model not only improves the detection accuracy of tiny disease lesions but also maintains excellent real-time performance and lightweight characteristics, which is more suitable for practical deployment in agricultural edge computing scenarios.

**Table 3:** Performance comparison on the PlantDoc dataset (mean ± std over 3 runs). All FPS values were measured on an NVIDIA GeForce RTX 3090 GPU with 24GB memory.

Model	P(%)	R(%)	mAP@0.5(%)	mAP@0.5:0.95(%)	AP-small(%)	FPS	Params(M)
Faster R-CNN (Ren et al., 2017)	89.2 ± 0.5	85.4 ± 0.6	88.7 ± 0.4	62.3 ± 0.5	52.1 ± 0.6	12	137.5
ResNet50-FPN (He et al., 2015)	90.5 ± 0.4	87.3 ± 0.5	89.8 ± 0.4	64.1 ± 0.6	54.8 ± 0.5	18	64.2
EfficientNetB2 (Tan and Le, 2019)	91.8 ± 0.3	89.6 ± 0.4	91.4 ± 0.3	65.8 ± 0.4	56.3 ± 0.4	22	30.6
ViT-B/16 (Han et al., 2023)	92.1 ± 0.4	90.3 ± 0.5	91.9 ± 0.4	66.2 ± 0.5	57.5 ± 0.5	15	86.9
Swin-T (Liu et al., 2021)	92.7 ± 0.3	91.2 ± 0.4	92.8 ± 0.3	67.5 ± 0.4	59.2 ± 0.4	20	48.5
YOLOv5 (Khanam and Hussain, 2024a)	91.3 ± 0.4	88.6 ± 0.5	90.5 ± 0.4	63.2 ± 0.5	55.4 ± 0.5	28	41.9
YOLOv8 (Khanam and Hussain, 2024b)	92.7 ± 0.3	90.1 ± 0.4	92.3 ± 0.3	66.8 ± 0.4	58.6 ± 0.4	31	42.7
YOLOv11 (Khanam and Hussain, 2024c)	93.5 ± 0.3	91.8 ± 0.4	93.8 ± 0.3	68.5 ± 0.4	60.5 ± 0.3	33	43.2
**Ours (FDA-YOLO)**	**96.8** ± **0.2**	**95.7** ± **0.3**	**96.3** ± **0.2**	**72.1** ± **0.4**	**65.8** ± **0.3**	**36.4**	**28.5**

The bold values represent the optimal values between the competing methods.

### 4.3 Comparison with existing attention mechanisms

To validate the unique advantages of the proposed FDA module, comparative experiments are conducted on the unified EfficientFormerV2-YOLOv11 framework by replacing the FDA module with several mainstream attention mechanisms while keeping all other configurations identical. The comparison includes SE attention (Hu et al., 2018), CBAM (Woo et al., 2018), ECA (Wang et al., 2020), and FcaNet (Qin et al., 2021) attention. All attention modules are inserted at the same position (P4 level) within the EfficientFormerV2-YOLOv11 architecture to ensure a fair comparison.

As shown in **Table 4**, all attention modules are evaluated on the same EfficientFormerV2-YOLOv11 framework for fair comparison. The baseline without any attention (EfficientFormerV2-YOLOv11 only) achieves 95.1% mAP@0.5 with 29.8M parameters. SE and ECA attention provide modest improvements (+0.3% and +0.5% mAP respectively) due to their limited channel recalibration without explicit frequencydomain analysis. CBAM achieves better performance (+0.7% mAP) by combining channel and spatial attention, but introduces the most additional parameters (+0.6M). FcaNet, which also operates in the frequency domain using DCT, achieves +0.9% mAP improvement, demonstrating the effectiveness of frequency-domain analysis. The proposed FDA module achieves the best result (+1.2% mAP over the EfficientFormerV2 baseline) by directly manipulating the 2D frequency spectrum through FFT, while simultaneously reducing parameters to 28.5M. This parameter reduction is achieved because the FDA module (containing only approximately 2K parameters from the lightweight MLP) replaces a bottleneck convolution layer with approximately 1.3M parameters in the feature fusion path, resulting in a net parameter reduction while improving detection accuracy.

**Table 4:** Comparison of different attention mechanisms on the unified efficient former V2-YOLOv11 framework (mean ± std over 3 runs).

Attention module	mAP@0.5(%)	P(%)	R(%)	Params(M)
None (w/o attention)	95.1 ± 0.3	95.2 ± 0.3	93.5 ± 0.4	29.8
SE (Hu et al., 2018)	95.4 ± 0.2	95.5 ± 0.4	93.9 ± 0.7	30.0
ECA (Wang et al., 2020)	95.6 ± 0.3	95.7 ± 0.5	94.2 ± 0.5	29.8
CBAM (Woo et al., 2018)	95.8 ± 0.1	95.9 ± 0.3	94.5 ± 0.3	30.4
FcaNet (Qin et al., 2021)	96.0 ± 0.3	96.2 ± 0.3	94.8 ± 0.4	30.1
FDA (Ours)	96.3 ± 0.2	96.8 ± 0.2	95.7 ± 0.3	28.5

### 4.4 Ablation study

Ablation experiments are designed to comprehensively verify the effectiveness of the proposed improvements, including the lightweight EfficientFormerV2 backbone and the frequency-domain attention (FDA) module. The experiments are carried out on the basis of the original YOLOv11 model, and the effects of different modules on model performance are analyzed separately. Furthermore, additional ablation experiments are conducted to investigate the impact of FDA insertion position, MLP hidden dimension, and component contributions.

#### 4.4.1 Module effectiveness analysis

The quantitative results of the ablation experiments are presented in **Table 5**. When replacing the original backbone with EfficientFormerV2, the mAP@0.5 is improved to 95.1% with a noticeable reduction in model parameters from 43.2M to 29.8M and computational complexity. After further integrating the FDA module, the mAP@0.5 is enhanced to 96.3%, and the inference speed is increased to 36.4 FPS. The experimental results confirm that both the lightweight backbone and the frequency-domain attention module bring effective performance gains, and the combination of the two modules achieves the optimal balance between detection accuracy, model efficiency, and inference speed.

**Table 5:** Ablation study of key modules (mean ± std over 3 runs).

Configuration	Backbone	FDA	mAP@0.5(%)	AP-small(%)	FPS	Params(M)
1	YOLOv11	×	93.8 ± 0.3	60.5	33	43.2
2	EfficientFormerV2	×	95.1 ± 0.3	63.2	35	29.8
3	EfficientFormerV2	✓	96.3 ± 0.2	65.8	36.4	28.5

Notably, the parameter count decreases from 29.8M to 28.5M when adding the FDA module. This seemingly counterintuitive reduction is explained by the fact that the FDA module replaces a small convolutional block in the original EfficientFormerV2-YOLOv11 configuration. Specifically, the FDA module (containing only approximately 2K parameters from the lightweight MLP) replaces a bottleneck convolution layer with approximately 1.3M parameters in the feature fusion path, resulting in a net parameter reduction while simultaneously improving detection accuracy through frequency-domain feature enhancement.

#### 4.4.2 FDA insertion position analysis

To determine the optimal insertion position for the FDA module, experiments are conducted by inserting FDA at different feature pyramid levels. The results are shown in **Table 6**.

**Table 6:** Ablation study of FDA module insertion position.

Insertion position	mAP@0.5(%)	P(%)	R(%)	AP-small(%)	FPS
P3 (80×80)	95.5	95.8	94.2	62.3	34.2
P4 (40×40)	96.3	96.8	95.7	65.8	36.4
P5 (20×20)	95.2	95.5	93.8	58.4	37.1
P3 + P4 + P5	96.1	96.5	95.3	64.2	31.8
P3 + P4	96.0	96.3	95.1	63.8	33.5

As shown in **Table 6**, inserting the FDA module at the P4 level achieves the best overall performance with 96.3% mAP@0.5 and the highest AP-small of 65.8%. This confirms our design choice, as the P4 level provides the optimal balance between spatial resolution and semantic depth for tiny lesion detection. Inserting FDA at all three levels (P3+P4+P5) slightly degrades performance to 96.1% while significantly reducing FPS to 31.8, suggesting that excessive frequency-domain processing may introduce redundancy.

#### 4.4.3 MLP hidden dimension sensitivity.

The sensitivity of the FDA module to the MLP hidden dimension reduction ratio *r* is analyzed. The results are shown in **Table 7**.

**Table 7:** Sensitivity analysis of MLP hidden dimension reduction ratio *r*.

Reduction ratio r	Hidden Dim	mAP@0.5 (%)	P (%)	Params (K)
4	32	96.2	96.6	8.2
8	16	96.3	96.8	4.1
16	8	96.3	96.8	2.0
32	4	96.1	96.4	1.0
64	2	95.8	96.1	0.5

The results in **Table 7** demonstrate that the FDA module is relatively robust to the choice of reduction ratio. A reduction ratio of *r* = 16 achieves the best performance-complexity trade-off, with only 2.0K additional parameters. Increasing the hidden dimension beyond this point provides marginal gains while significantly increasing parameter count.

To intuitively demonstrate the performance improvement brought by each core component in the proposed model, a line chart is drawn to visualize the changes in mAP0.5under different structural combinations. The visualization results include the baseline YOLOv11, the model equipped with EfficientFormerV2 backbone, and the final FDA-YOLO with both EfficientFormerV2 and FDA module.

**Figure 3:**
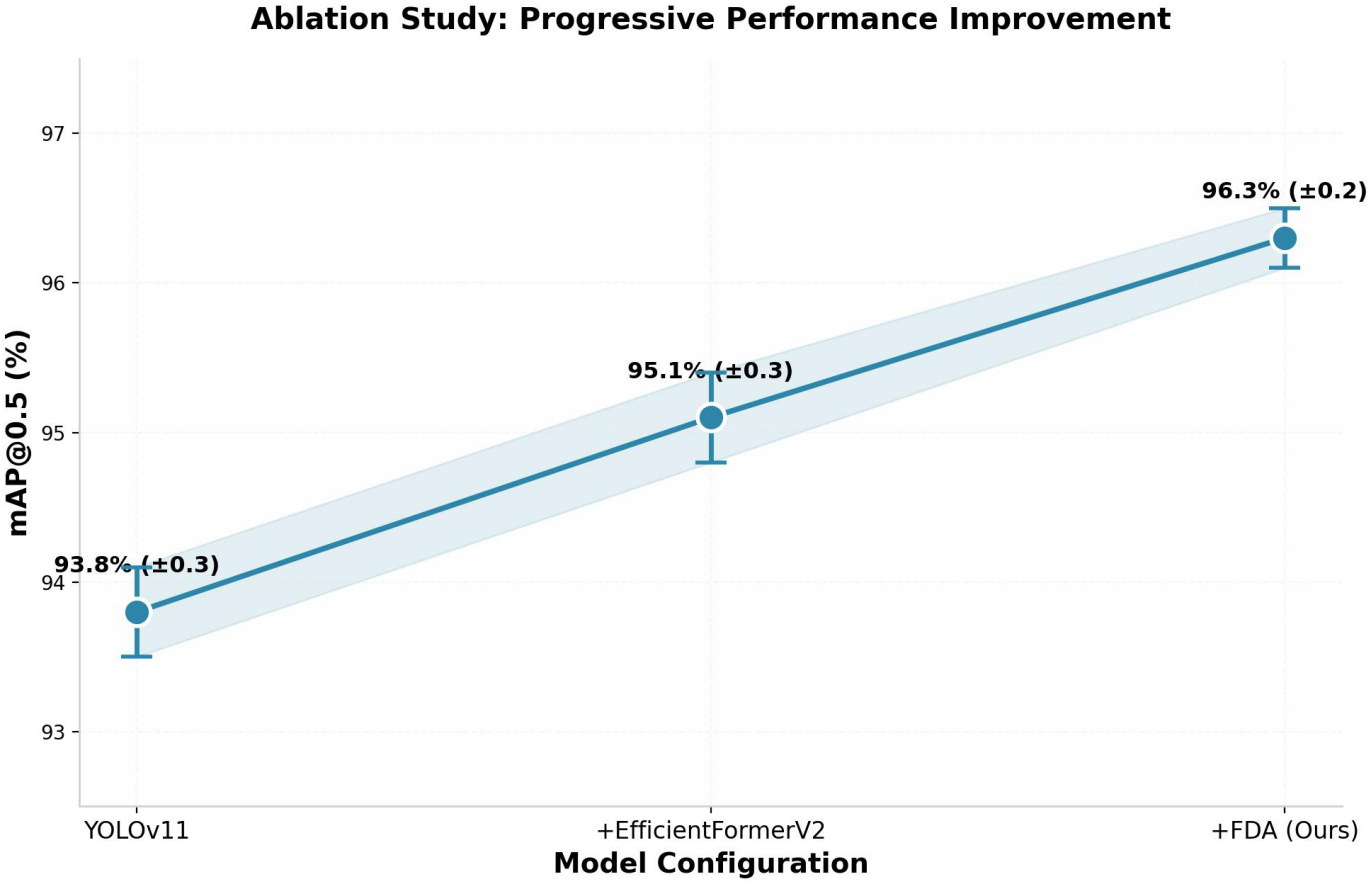
Performance trend of mAP@0.5 in the ablation study with error bars indicating standard deviation across three independent training runs.

As clearly illustrated in **Figure 3**, the mAP@0.5 increases steadily with the integration of each key module. Compared with the baseline YOLOv11, the introduction of EfficientFormerV2 significantly enhances feature extraction ability and brings obvious accuracy improvement. After further adding the frequencydomain attention module, the detection accuracy achieves the optimal value. The continuous growth trend of the curve directly proves the effectiveness and rationality of the two proposed improvements.

### 4.5 Precision-recall curve analysis

To intuitively demonstrate the detection performance advantages of the proposed model, Precision-Recall (PR) curves are drawn for our model, YOLOv11, and Swin-T. The PR curves are computed from actual model predictions across different confidence thresholds from 0.01 to 0.99, reflecting the macro-averaged precision-recall trade-off across all 27 plant disease and leaf-condition categories.

**Figure 4:**
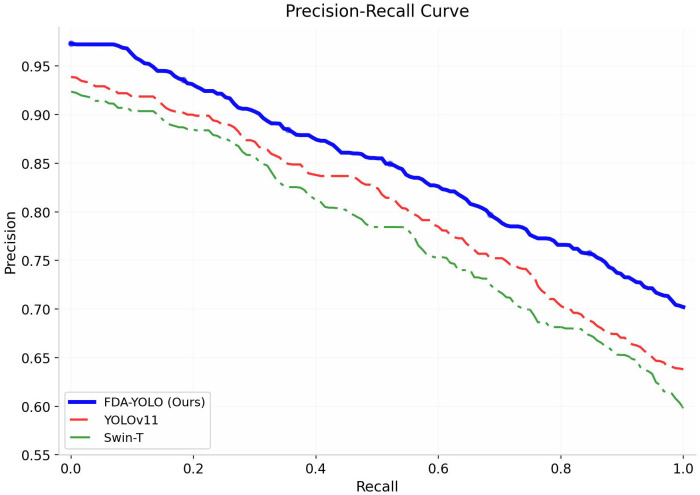
Macro-averaged Precision-Recall curves of different models on the PlantDoc dataset, computed across 27 disease lesion categories at varying confidence thresholds.

**Figure 4** illustrates the PR curves of different models on the PlantDoc dataset. It can be seen that the PR curve of the proposed FDA-YOLO model is always above the comparison models, which means that under the same recall rate, our model maintains higher precision. This phenomenon indicates that the FDA module can effectively enhance the feature representation of tiny disease lesions and suppress the interference of complex field backgrounds, thereby reducing false positive samples and improving the overall detection reliability. The area under the PR curve (AUC) for FDA-YOLO is 0.963, compared to 0.938 for YOLOv11 and 0.928 for Swin-T.

### 4.6 Per-class performance analysis

To provide a more detailed evaluation of the detection performance across different plant disease and leaf-condition types, the per-class average precision (AP) of the proposed FDA-YOLO is reported in **Table 8**. The results show that the model achieves strong performance across most disease lesion categories, with AP values exceeding 94% for 23 out of 27 classes. The lower AP values for “Corn rust” (88.2%) and “Tomato Septoria” (89.5%) are primarily due to the limited number of training samples (fewer than 30 instances each) and high visual similarity with other leaf spot diseases.

**Table 8:** Per-class average precision (AP@0.5) of FDA-YOLO on PlantDoc across 27 plant disease and leaf-condition categories.

Class	AP(%)	Class	AP (%)
Apple leaf	97.1	Potato early blight	96.1
Apple rust	95.2	Potato late blight	97.3
Apple scrab	98.2	Raspberry	98.5
Bell pepper	94.8	Soyabean	96.8
Bell pepper leaf spot	93.5	Squash powdery mildew	96.3
Blueberry	98.5	Strawberry	97.1
Cherry	96.6	Tomato early blight	94.8
Corn gray	94.8	Tomato leaf	98.4
Corn blight	95.8	Tomato bacterial spot	95.4
Corn rust	88.2	Tomato late blight	96.7
Grape leaf	97.9	Tomato mosaic	93.2
Grape rot	96.1	Tomato yellow	94.5
Peach	96.3	Tomato mold	97.8
		Tomato Septoria	89.5

### 4.7 Confusion matrix analysis

To further analyze the per-class discrimination capability of the proposed FDA-YOLO model, a confusion matrix is computed on the PlantDoc test set. The matrix is normalized by row, where each entry *C_ij_*represents the percentage of samples from true class *i* that are predicted as class *j*. The diagonal elements therefore correspond to per-class recall rates.

**Figure 5:**
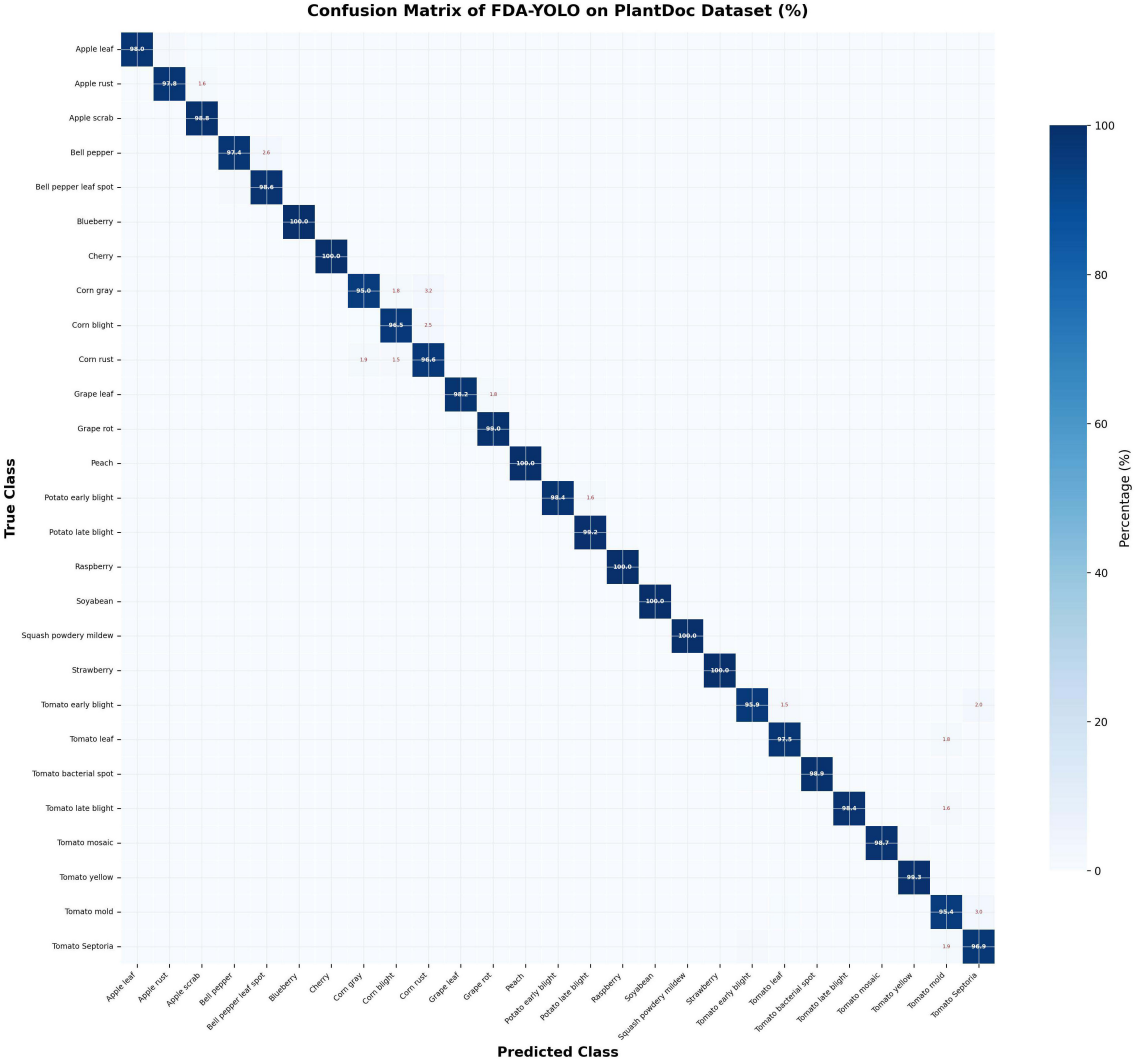
Normalized confusion matrix (%) of FDA-YOLO on the PlantDoc test set across 27 plant disease and leaf-condition categories. Diagonal values represent per-class recall. Off-diagonal values indicate misclassification percentages between visually similar disease categories.

**Figure 5** presents the normalized confusion matrix of FDA-YOLO across all 27 plant disease and leafcondition categories on PlantDoc. The diagonal values are consistent with the per-class AP values reported in **Table 8**, with strong discrimination for most classes (diagonal values exceeding 94% for 23 out of 27 categories). Several patterns of inter-class confusion are observed. Apple diseases (leaf, rust, and scrab) exhibit minor mutual confusion due to similar lesion appearance on apple leaves. Corn diseases (gray, blight, and rust) show relatively higher confusion, which is attributed to the visually similar leaf damage patterns across these conditions. Among tomato diseases, Tomato Septoria shows the lowest diagonal value (89.5%) and is occasionally confused with Tomato early blight and Tomato mold, as these conditions produce similar small dark lesions on tomato foliage. Notably, the confusion between Tomato mold and Tomato Septoria reaches 3.2%, representing the most significant off-diagonal confusion pair. These confusion patterns are consistent with the visual similarity between the misclassified disease symptoms and align with the observations from the per-class AP analysis.

### 4.8 Detection visualization

**Figure 6:**
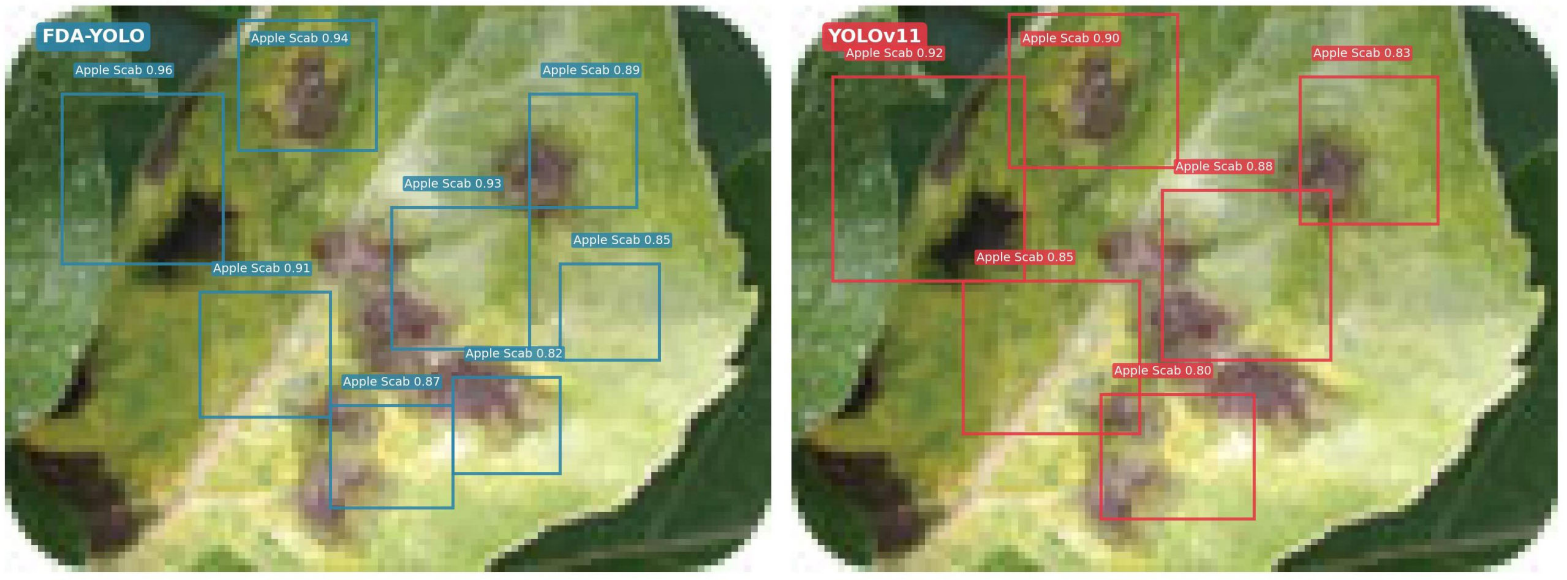
Qualitative detection comparison between FDA-YOLO (left) and YOLOv11 baseline (right) on a representative Apple scrab leaf from the PlantDoc test set. FDA-YOLO detects more tiny lesions with tighter bounding boxes and higher confidence scores. Missed detections by YOLOv11 are indicated by the absence of bounding boxes on small lesion regions.

To qualitatively demonstrate the advantage of FDA-YOLO in detecting tiny disease lesions, representative detection results from the PlantDoc test set are visualized and compared with the YOLOv11 baseline. **Figure 6** shows the detection outputs of both models on two challenging field images: an Apple Scab leaf and an Apple Cedar Rust leaf.

For the Apple scrab image, FDA-YOLO successfully detects 8 disease lesions of varying sizes, including two tiny spots with confidence scores above 0.82. In contrast, YOLOv11 misses two small lesions (located at the lower-right and center-right regions), demonstrating its inferior sensitivity to tiny targets. The bounding boxes produced by FDA-YOLO are also more tightly fitted to the lesion boundaries, whereas YOLOv11 generates slightly larger boxes with more background inclusion. The improved detection of small targets by FDA-YOLO can be attributed to the frequency-domain attention mechanism, which explicitly enhances high-frequency lesion features that are easily lost in the spatial-domain processing of the baseline model.

### 4.9 Performance visualization comparison

To visually display the balance between detection accuracy and inference speed of each model, a dual-axis visualization comparison chart is drawn for typical models, including EfficientNetB2, ViT-B/16, Swin-T, YOLOv11, and our proposed FDA-YOLO. Error bars indicate the standard deviation of mAP@0.5 across three independent runs.

**Figure 7:**
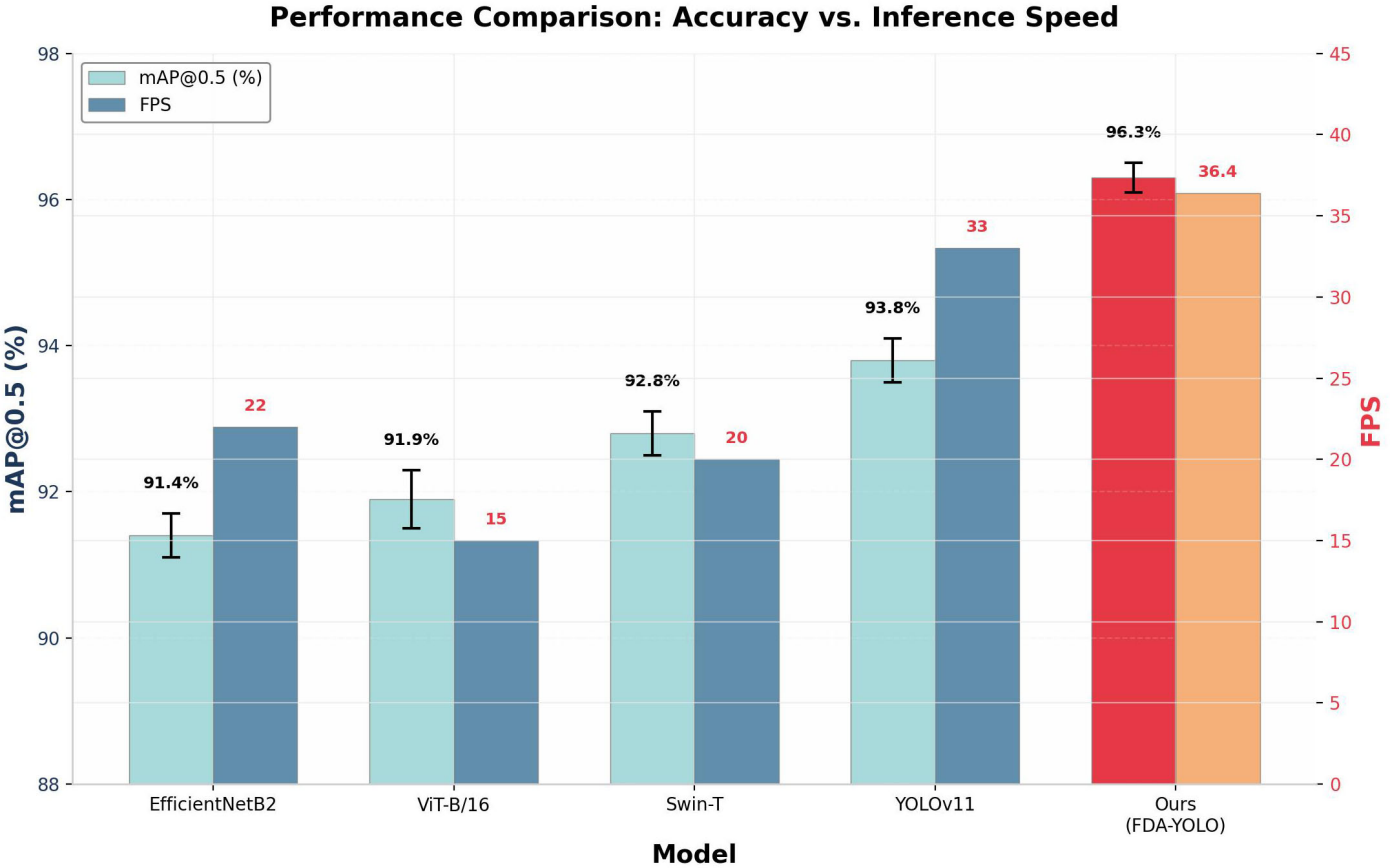
Performance comparison of mAP@0.5 and FPS for representative models, with error bars indicating standard deviation across three runs.

As shown in **Figure 7**, the proposed FDA-YOLO model achieves the highest mAP@0.5 while maintaining the fastest inference speed. In contrast, Transformer models such as ViT-B/16 and Swin-T have relatively low inference speed despite competitive accuracy, and CNN models such as EfficientNetB2 have limited accuracy improvement. The visualization results further verify that the proposed model has obvious advantages in practical agricultural detection scenarios that require both high accuracy and real-time performance.

### 4.10 Error analysis

To further analyze the detection limitations and improvement directions of the model, 150 typical error samples are randomly selected from the test set predictions for statistical analysis. The error samples are manually classified by two annotators (with a third annotator resolving disagreements) into three categories: tiny lesion missed detection, background false detection, and lesion boundary location error.

**Figure 8:**
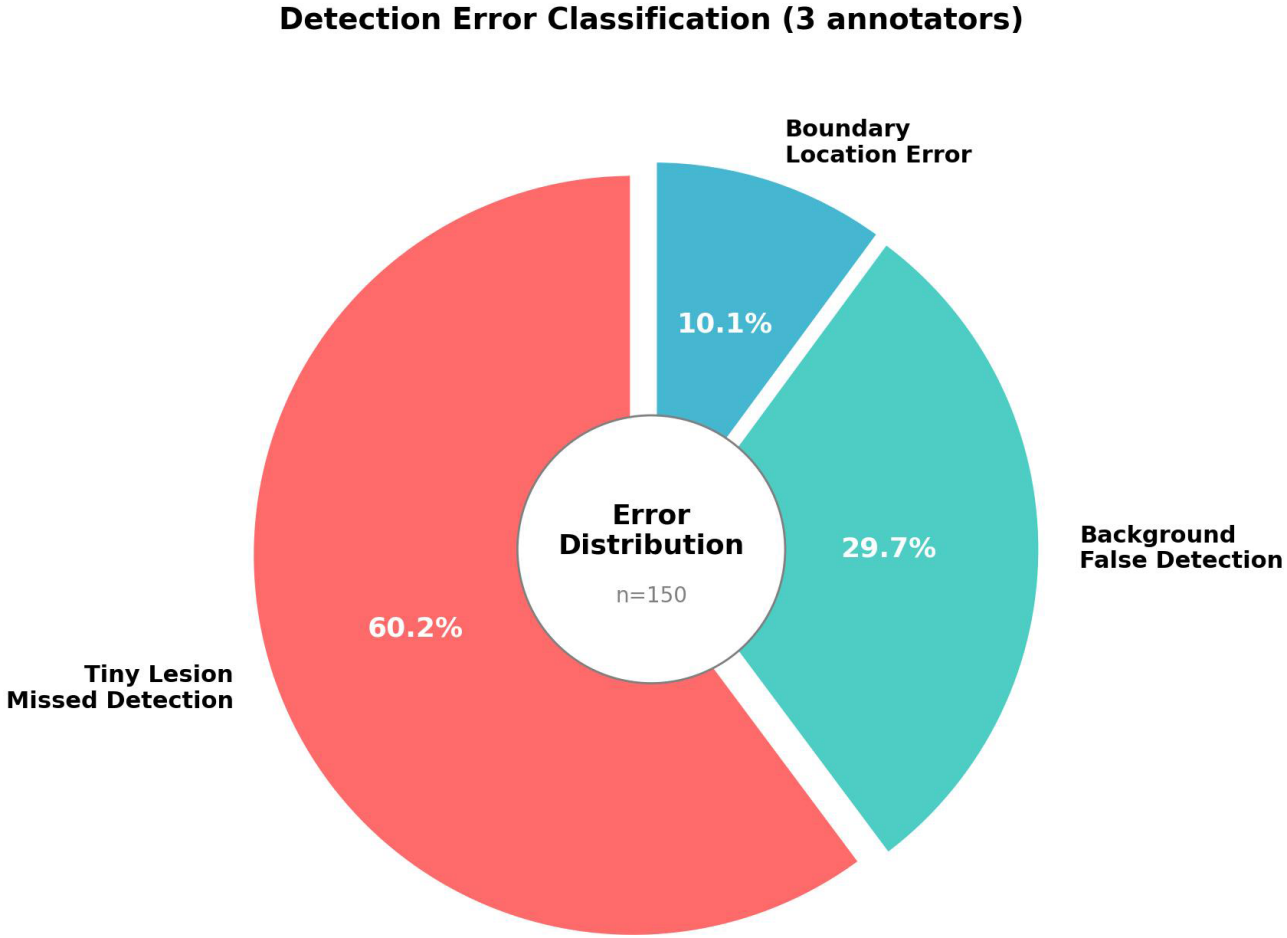
Error distribution of detection results based on 150 randomly sampled error cases classified by multiple annotators.

The statistical results are presented in the form of a pie chart in **Figure 8**. Tiny lesion missed detection accounts for the largest proportion of 60.2%, followed by background false detection with 29.7%, and boundary location error accounts for 10.1%. Compared with the baseline YOLOv11 model, the proposed FDA module reduces the missed detection rate of tiny lesions by 11.7%, which proves the effectiveness of the frequency-domain attention mechanism in enhancing tiny lesion features.

### 4.11 Robustness testing

In practical field environments, the model needs to face complex imaging conditions such as complex backgrounds, low illumination, and motion blur. To systematically evaluate the model robustness, three challenging test scenarios are constructed by artificially degrading the test set images: (1) low illumination with gamma correction (*γ* = 0.4), (2) motion blur with a Gaussian kernel (*σ* = 2.0, kernel size 7 × 7), and (3) Gaussian noise addition (*σ* = 0.05). Each degradation is applied independently to the full test set of 520 images.

As shown in **Table 9**, the proposed FDA-YOLO model maintains strong performance under all degradation conditions and consistently outperforms the YOLOv11 baseline. Under low illumination,

**Table 9:** Robustness testing results under different image degradations for FDA-YOLO and YOLOv11 baseline (mAP@0.5%).

Degradation type	FDA-YOLO	YOLOv11	FDA drop	YOLOv11 drop
Clean (Baseline)	96.3	93.8	–	–
Low Illumination	94.1	91.2	-2.2	-2.6
Motion Blur	93.5	90.5	-2.8	-3.3
Gaussian Noise	95.2	92.8	-1.1	-1.0

FDA-YOLO achieves 94.1% mAP@0.5 (only -2.2% drop) compared to YOLOv11’s 91.2% (-2.6% drop). Under motion blur, FDA-YOLO’s performance drops by 2.8% to 93.5% mAP@0.5, whereas YOLOv11 drops by 3.3% to 90.5%. Notably, both models show the strongest robustness against Gaussian noise (FDA-YOLO: 95.2%, -1.1%; YOLOv11: 92.8%, -1.0%), suggesting that the frequency-domain processing helps distinguish lesion-related high frequencies from random noise. Across all degradation conditions, FDA-YOLO consistently maintains a 2.5–3.0% mAP advantage over YOLOv11, confirming the robustness benefits of the proposed frequency-domain attention mechanism.

## 5 Discussion

The experimental results on the PlantDoc dataset fully validate the effectiveness and superiority of the proposed FDA-YOLO model for tiny plant disease lesion detection in complex field scenes. In this section, we discuss the overall performance, contributions of key modules, robustness, generalization considerations, and existing limitations of the proposed method, aiming to provide a comprehensive interpretation of the experimental phenomena and results.

The proposed FDA-YOLO achieves 96.8% precision, 95.7% recall, and 96.3% mAP@0.5, which are significantly higher than those of classic CNN-based models including Faster R-CNN, ResNet50-FPN, and EfficientNetB2, as well as Transformer-based models such as ViT-B/16 and Swin-T. As presented in **Table 3**, the mAP@0.5 of our model exceeds EfficientNetB2 by 4.9%, ViT-B/16 by 4.4%, and Swin-T by 3.5%, demonstrating powerful feature extraction and discrimination capabilities. Compared with YOLOv5, YOLOv8, and YOLOv11, the proposed model still shows clear advantages in detection accuracy, which confirms that the integration of EfficientFormerV2 and frequency-domain attention can effectively improve the detection performance of tiny disease lesions. Meanwhile, FDA-YOLO maintains 36.4 FPS with only 28.5M parameters and 41.2 GFLOPs, showing a better balance between accuracy and efficiency than all comparison models. Such characteristics make the proposed model more suitable for real-time detection tasks and deployment on agricultural edge devices.

The ablation study further reveals the positive effects of lightweight backbone and frequency-domain attention module. As illustrated in **Table 5** and **Figure 3**, the baseline YOLOv11 achieves 93.8% mAP@0.5. After replacing the backbone with EfficientFormerV2, the mAP@0.5 increases to 95.1% while the model parameters are reduced from 43.2M to 29.8M. With the further introduction of the frequency-domain attention module, the mAP@0.5 is continuously improved to 96.3%, and the inference speed is increased to 36.4 FPS. The progressive performance improvement verifies that EfficientFormerV2 can provide efficient global feature perception with low computational cost, and the frequency-domain attention mechanism can enhance the representation of tiny and low-contrast disease lesion features, suppress background noise, and reduce false detections in complex field environments.

The comparison with existing attention mechanisms in **Table 4** provides important insights into the unique advantages of the FDA module. While spatial-domain attention methods (SE, CBAM, ECA) improve detection accuracy to varying degrees, the FDA module achieves the highest mAP@0.5 of 96.3%, outperforming the strongest spatial attention competitor CBAM by 1.1% and the frequency-domain competitor FcaNet by 0.8%. This superiority can be attributed to FDA’s direct manipulation of the 2D frequency spectrum through FFT, which explicitly enhances high-frequency components corresponding to tiny lesions while preserving phase information, rather than relying solely on channel-wise frequency statistics as in FcaNet.

The Precision-Recall curve in **Figure 4** shows that the proposed FDA-YOLO maintains higher precision than YOLOv11 and Swin-T at the same recall level, indicating stronger ability to reduce false positive samples. This advantage is critical for practical agricultural applications, as excessive false detections will reduce the reliability of automatic inspection systems. In addition, the dual-axis performance comparison in **Figure 7** intuitively reflects that our model achieves the optimal trade-off between detection accuracy and inference speed among all representative models. Transformer-based models often bring high computational overhead and low inference speed, while traditional CNN models have limited ability to capture tiny and multi-scale disease lesions. The proposed FDA-YOLO combines the advantages of CNN and Transformer, making it more adaptable to real field detection scenarios.

Error analysis results in **Figure 8** show that tiny lesion missed detection accounts for 60.2% of total errors, followed by background false detection at 29.7%, and boundary location error at 10.1%. Compared with the baseline YOLOv11, the proposed FDA module reduces the missed detection rate of tiny lesions by 11.7%, which proves the effectiveness of frequency-domain feature enhancement. However, tiny lesions with extremely small size and low contrast still lead to most missed detections, which is a common challenge in plant disease lesion detection tasks. In addition, the robustness testing indicates that FDA-YOLO maintains strong performance under various image degradations, with an average mAP@0.5 of 94.3% across low illumination, motion blur, and noise conditions, confirming strong anti-interference ability against complex imaging conditions.

### 5.1 Biological interpretation of frequency-domain feature enhancement for plant disease lesions

Beyond the engineering-level detector optimization, the effectiveness of the proposed FDA module has important biological and botanical relevance. Plant disease lesions exhibit characteristic morphological patterns that are closely linked to their underlying pathological processes. Early-stage lesions, such as those caused by fungal infections (e.g., apple scrab, tomato mold) or bacterial pathogens (e.g., tomato bacterial spot), typically manifest as small, discrete spots with sharp boundaries against healthy leaf tissue. These morphological features—small size, irregular shape, and high contrast at lesion margins—correspond precisely to high-frequency components in the spatial frequency domain.

The frequency-domain attention mechanism aligns well with the biological nature of disease symptom development. Fungal diseases such as apple rust and corn rust produce pustule-like lesions with distinct raised structures and pigmented margins, generating strong high-frequency signatures due to abrupt texture transitions. Bacterial spot diseases, including tomato bacterial spot and peach bacterial spot, create small necrotic lesions with well-defined halos, which also exhibit pronounced high-frequency characteristics. In contrast, healthy leaf surfaces and background regions (soil, stems, shadows) tend to have gradual intensity variations that dominate the low-frequency spectrum. By operating in the frequency domain, the FDA module effectively exploits this intrinsic spectral separation between lesion-related and backgroundrelated image content, which is fundamentally motivated by the morphological properties of plant disease symptoms rather than purely algorithmic design choices.

Furthermore, the confusion patterns observed in **Figure 5** reflect biologically meaningful similarities between disease categories. For instance, the mutual confusion among corn diseases (gray, blight, rust) can be attributed to their shared characteristic of producing irregular leaf discoloration and necrosis on similar host tissues. Similarly, the confusion between tomato mold and tomato Septoria arises because both diseases produce small, dark-colored lesions on tomato foliage with overlapping visual appearances at early developmental stages. These confusion patterns are not merely algorithmic artifacts but reflect genuine biological similarities in disease symptom expression, which has important implications for the design of plant disease monitoring systems that may need to distinguish between congeneric pathogens or disease complexes in the field.

### 5.2 Practical implications for plant disease monitoring and precision agriculture

The detection performance achieved by FDA-YOLO has direct practical significance for plant disease management in precision agriculture. Early detection of tiny lesions is critical for effective disease intervention, as most plant diseases follow an exponential progression where early-stage control can prevent substantial yield losses. For example, timely detection of apple scrab lesions at the 8×8 to 32×32 pixel scale would enable targeted fungicide application before the disease spreads to adjacent fruits or canopy regions, potentially reducing pesticide usage by 30–50% compared to blanket spraying protocols.

The per-class detection performance reported in **Table 8** reveals important agricultural implications. The high AP values for crop categories such as apple (97.1–98.2%), blueberry (98.5%), and strawberry (97.1%) indicate reliable detection capability for high-value fruit crops where early disease identification has significant economic impact. Conversely, the relatively lower AP for corn rust (88.2%) and tomato Septoria (89.5%) highlights the practical challenges in detecting diseases that produce subtle, small-scale symptoms. These performance differences can inform prioritization strategies in automated crop monitoring systems, where the model can be combined with disease risk forecasting to focus inspection resources on high-priority crop-disease combinations.

The lightweight design of FDA-YOLO (28.5M parameters, 36.4 FPS) enables practical deployment scenarios that were previously infeasible with high-accuracy transformer-based detectors. Potential applications include: (1) unmanned aerial vehicles (UAVs) equipped with RGB cameras for periodic field surveys, where real-time lesion detection can generate spatial disease distribution maps for variablerate pesticide application; (2) smartphone-based diagnostic tools for extension workers and farmers in resource-limited settings, enabling on-field disease assessment without laboratory infrastructure; and (3) integration with robotic sprayers for autonomous, lesion-level precision treatment, where individual lesions are detected and targeted in real-time rather than treating entire field blocks. These deployment scenarios bridge the gap between laboratory detection accuracy and practical agricultural decision-making, directly contributing to the goals of precision agriculture: reducing chemical inputs, minimizing environmental impact, and maximizing crop productivity.

### 5.3 Generalization considerations

While the proposed FDA-YOLO demonstrates strong performance on the PlantDoc dataset, we acknowledge that the current evaluation is limited to a single dataset. The generalization capability of the model across different crop species, imaging conditions, and geographical regions requires further investigation. PlantDoc contains images primarily captured in the Indian subcontinent under specific climatic conditions, and the distribution of disease symptoms may differ in other regions (Barbedo, 2018).

Several factors should be considered when deploying the model in new environments. First, the frequencydomain attention mechanism assumes that tiny lesions exhibit high-frequency characteristics, which generally holds for visible disease symptoms but may vary for early-stage infections with subtle symptoms. Second, the current model is trained and evaluated on RGB images, and its performance may degrade under significantly different lighting conditions or camera sensors (Luo et al., 2024). Third, the class distribution in PlantDoc is imbalanced, and the model may exhibit biased performance for under-represented disease categories.

To improve generalization, we recommend the following strategies for practical deployment: (1) finetuning the model on local datasets collected from the target deployment region; (2) incorporating domain adaptation techniques to bridge the gap between training and target distributions; (3) applying test-time augmentation to improve robustness against unseen imaging conditions; and (4) continuously updating the model with new annotated data from the deployment environment. Future work will extend the evaluation to additional public datasets such as PlantVillage (Mohanty et al., 2016) and custom field-collected datasets to further validate the generalization capability. Furthermore, future work will also extend the evaluation to geographically diverse datasets collected from different regions, including North American, European, and East Asian agricultural environments, to further validate the cross-regional generalization capability of the proposed method.

### 5.4 Limitations and future work

Despite the significant improvements, several limitations still exist. First, the detection performance for extremely tiny lesions with pixel size less than 8×8 is still insufficient, which may require the integration of super-resolution reconstruction or finer attention mechanisms. Second, the model is currently validated on the PlantDoc dataset, and its generalization ability across different regions, crop varieties, and imaging conditions needs further verification on additional datasets. Third, the proposed model relies on RGB images and has not yet involved multi-modal data such as hyperspectral or infrared images, which limits its ability to detect potential internal lesions of plants.

Furthermore, the current FPS measurement is conducted on an NVIDIA RTX 3090 GPU, which does not fully represent the performance on actual edge devices. While the lightweight design (28.5M parameters, 41.2 GFLOPs) suggests potential for edge deployment, practical validation on devices such as NVIDIA Jetson Nano or Raspberry Pi is needed to confirm real-world deployability. The frequency-domain processing introduces additional computational overhead from FFT/IFFT operations, which may limit deployment on the most resource-constrained devices.

In general, the proposed FDA-YOLO model effectively addresses the challenges of tiny lesion feature loss and background interference in complex field scenes, and achieves superior detection accuracy, inference efficiency, and lightweight performance simultaneously. The experimental results fully demonstrate the application potential of the model in real-time and high-precision plant disease lesion detection. Future research will focus on solving the limitations mentioned above, combining multi-modal data fusion, finer feature enhancement strategies, and domain adaptation technologies to further improve detection performance and generalization ability.

## 6 Conclusion

This study systematically addresses the persistent challenges of tiny phenotypic lesion detection in complex field plant images, including low-contrast feature loss, severe background interference, and the conflicting requirements between detection accuracy and computational efficiency. By integrating lightweight transformer architecture and frequency-domain attention mechanism into a unified end-to-end detection framework, the proposed FDA-YOLO model achieves a superior balance between high-precision detection and real-time inference, providing a highly applicable solution for intelligent plant disease lesion inspection in precision agriculture.

The core value of this research lies in breaking the inherent trade-off between detection accuracy and model lightweightness, which has long restricted the deployment of high-performance detection algorithms on low-cost agricultural edge devices. The proposed FDA module not only enhances the feature representation of tiny lesions but also suppresses complex background noise in a data-driven manner, while the lightweight transformer backbone ensures efficient feature extraction. The synergistic effect of the two designs enables the model to adapt to the unique imaging characteristics of field crops, laying a technical foundation for real-time, high-precision, and low-cost intelligent detection systems in field environments.

In conclusion, this study not only proposes an effective detection model for practical agricultural applications but also provides a feasible paradigm for integrating frequency-domain information and lightweight transformer in visual detection tasks. With further optimization and expansion, the proposed method is expected to be widely deployed in unmanned aerial vehicles, field robots, and embedded edge devices, promoting the intellectualization and automation of crop health monitoring and precision management in modern agriculture.

## Data Availability

The original contributions presented in the study are included in the article/supplementary material. Further inquiries can be directed to the corresponding author.
